# Corrosion Initiation and Propagation on Carburized Martensitic Stainless Steel Surfaces Studied via Advanced Scanning Probe Microscopy

**DOI:** 10.3390/ma12060940

**Published:** 2019-03-21

**Authors:** Armen Kvryan, Corey M. Efaw, Kari A. Higginbotham, Olivia O. Maryon, Paul H. Davis, Elton Graugnard, Hitesh K. Trivedi, Michael F. Hurley

**Affiliations:** 1 Micron School of Materials Science & Engineering, Boise State University, Boise, ID 83725-2090, USA; armenkvryan@u.boisestate.edu (A.K.); coreyefaw@u.boisestate.edu (C.M.E.); karilivingston@u.boisestate.edu (K.A.H.); oliviamaryon@u.boisestate.edu (O.O.M.) pauldavis2@boisestate.edu (P.H.D.); eltongraugnard@boisestate.edu (E.G.); 2 UES, Inc., 4401 Dayton Xenia Rd, Dayton, OH 45432, USA; hitesh.trivedi.ctr@us.af.mil

**Keywords:** corrosion, bearing steels, martensitic stainless steel, aerospace, atomic force microscopy (AFM), scanning Kelvin probe microscopy (SKPFM), nanoscale, electrochemistry, wear, Pyrowear 675/AMS 5930

## Abstract

Historically, high carbon steels have been used in mechanical applications because their high surface hardness contributes to excellent wear performance. However, in aggressive environments, current bearing steels exhibit insufficient corrosion resistance. Martensitic stainless steels are attractive for bearing applications due to their high corrosion resistance and ability to be surface hardened via carburizing heat treatments. Here three different carburizing heat treatments were applied to UNS S42670: a high-temperature temper (HTT), a low-temperature temper (LTT), and carbo-nitriding (CN). Magnetic force microscopy showed differences in magnetic domains between the matrix and carbides, while scanning Kelvin probe force microscopy (SKPFM) revealed a 90–200 mV Volta potential difference between the two phases. Corrosion progression was monitored on the nanoscale via SKPFM and in situ atomic force microscopy (AFM), revealing different corrosion modes among heat treatments that predicted bulk corrosion behavior in electrochemical testing. HTT outperforms LTT and CN in wear testing and thus is recommended for non-corrosive aerospace applications, whereas CN is recommended for corrosion-prone applications as it exhibits exceptional corrosion resistance. The results reported here support the use of scanning probe microscopy for predicting bulk corrosion behavior by measuring nanoscale surface differences in properties between carbides and the surrounding matrix.

## 1. Introduction

The performance of advanced gas turbine engines is currently limited by degradation of the mechanical components, in particular, rolling bearing elements, such as the raceway [1]. This is because aerospace engine bearings are subject to extreme operating conditions, including elevated temperatures, high speeds, vibratory stresses, rolling contact fatigue, and complex lubricant and environment interactions [2]. Accordingly, both high hardness and high toughness are critical requirements for aerospace bearing materials, yet achieving both in a single material is challenging. M50, a through-hardened carbon steel, was developed for aircraft engine bearing applications and has become the standard bearing steel used in the United States due to its ability to perform well at high temperatures while maintaining relatively high fracture toughness compared to earlier generation carbon steels, such as AISI 52100 (UNS G52986) [1,3,4]. In the case of sea-based or coastal aircraft operations however, open turbine engine systems can limit the ability of ester-based lubricants to provide wear and corrosion protection, as the surrounding environment introduces water and marine aerosols into the engine during both storage and operation [5]. The presence of water in the lubricant can then serve to initiate aqueous corrosion during engine cycling and downtime [5]. Consequently, current aero-engine performance is limited by corrosion-enhanced wear of the metallic bearings and drive components, which leads to increased maintenance and premature failure [1,6,7,8]. Thus, there has been significant research effort to develop alternative bearing steels to M50 that exhibit enhanced corrosion resistance to support increased engine performance [3,4,7,8,9,10].

Martensitic stainless steels (MSSs) were developed for use in applications where high wear resistance and toughness is required whilst maintaining high corrosion resistance. These properties, combined with their potential for high hardness upon heat treatment [1,11,12,13,14,15], have led to MSSs being implemented in many demanding applications, including bearings, molds, nuclear reactors, hydroelectric engines, and petrochemical steam and gas turbines and buckets [1,11,12,13,14,15,16,17,18,19,20]. To improve surface wear resistance while maintaining the corrosion resistance of the core, MSSs can instead be surface treated (carburized), with carbon incorporated into the sample surface at elevated temperatures to form hard carbides with alloying elements such as chromium or vanadium [1,21,22,23].

Highly corrosion-resistant MSSs (e.g., Cronidur 30 or XD15NW) include additions of alloying elements (and/or nitrogen) and can have poor adhesive and wear performance [24]. While not as corrosion resistant, UNS S42670 or AMS 59030B (referred to herein as P675) are relatively cost-efficient MSSs with high corrosion resistance (equivalent to 440C steel) and bulk fracture toughness (higher than M50) [25]. P675 was specifically engineered for aerospace bearing applications in advanced gas-turbine engines, where conventional bearing steels (e.g., M50 and 440C) are adversely affected by corrosion in aggressive environments and/or do not have sufficient high temperature wear performance [8]. Although P675 shows improvement in corrosion resistance relative to conventional bearing steels, higher surface hardness would lead to a longer wear lifetime in-service. Accordingly, secondary surface processing has been targeted as a way to increase the hardness and wear resistance of P675 [7,9,10,26]. Such surface treatments impart a graded microstructure that extends ~1000 µm below the metal surface. Optimized wear properties are obtained by balancing the surface hardness and core ductility of composite microstructures across the gradient region. However, the increased surface hardness typically comes at the expense of corrosion resistance, as the formation of carbides on the surface locally depletes corrosion-resistant elements (e.g., chromium, vanadium, molybdenum) from the surrounding matrix [7,20,22,23,27,28].

The corrosion performance of various P675 surface treatments has been previously assessed through accelerated DC and AC electrochemical testing in aqueous solutions [7,9,10]. These investigations provided a ranking of corrosion performance, showing that the final tempering temperature and processing atmosphere had a considerable influence on both the overall corrosion rate and damage morphology. Compared to M50, surface hardened P675 can be significantly more corrosion-resistant, and higher processing temperatures typically increased susceptibility to general corrosion damage, while lower temperatures exhibited more localized corrosion relative to untreated P675 [7]. The influence of processing on P675 wear performance for the same steels in non-corrosive wear testing has also been reported, where higher processing temperatures (HTT) yielded longer bearing lifetimes compared to low-temperature temper (LTT) [29,30]. However, there remains a need for research into the interdependency between simultaneously balancing corrosion resistance and surface hardness for bearing applications, since wear resistance (i.e., bearing performance) in corrosive environments is ultimately limited by corrosion [11].

Investigation of surface electronic properties can provide information to aid in the prediction of corrosion initiation sites [31]. Recently, scanning Kelvin probe force microscopy (SKPFM) has been used to investigate the role of nano- and micro-scale surface features on corrosion behavior [19,32,33,34,35,36,37,38,39,40,41,42]. Additionally, magnetic force microscopy (MFM) [43,44,45] has been used to similarly provide insight into the magnetic behavior of alloy surfaces. SKPFM permits measurement with nanoscale resolution of Volta potential differences (VPDs), which are related to the electronic work function (EWF), while MFM provides information regarding the magnitude and orientation of the magnetic moments of surface domains. Likewise, in situ atomic force microscopy (AFM) has been used to monitor morphological changes during corrosion in electrolyte solution and link them to the electrochemical behavior of the material [19,46,47,48,49]. The current work presents the first application of such techniques to investigate corrosion behavior of MSS P675 with various surface treatments. Since corrosion is the most common precursor to wear damage during aero-engine operation [8], the time to onset and rate of corrosion can directly control maintenance requirements and operational costs. Initiation and propagation are critical considerations because they determine both wear behavior as well as the lifetime of the part or engine [8,50,51]. The focus of this study is to understand the effects of heat treatment processing parameters on corrosion evolution in P675 by utilizing a combination of scanning probe microscopy (SPM) techniques and accelerated corrosion testing, thereby linking surface microstructural differences (on the nanoscale) with observed macroscale surface corrosion behavior and wear performance.

## 2. Materials and Methods

### 2.1. Materials

The nominal bulk composition of P675 (UNS S42670, the MSS studied here) prior to heat treatment is shown in Table 1 [29]. To increase surface hardness, P675 samples were carburized, followed by quenching and tempering, to harden the outer layer or case. Samples were cylindrical (9.5 mm diameter × 12 mm height) with post-treatment case depths of 750–1250 µm radially inward [9]. Samples differed in the final tempering temperature and carburization atmosphere: high-temperature tempering (HTT) at 496 °C, low-temperature tempering (LTT) at 315 °C, and carbo-nitrided (CN) where the case was obtained through a carburizing cycle followed by nitriding cycle during heat treating. Further details on the processing routes are discussed in previous works [9,10,29,30]. Prior to SPM characterization, samples were mechanically ground with SiC paper (to 2000 grit) in deionized (DI) water, followed by sequential polishing to 0.02 µm with a colloidal silica aqueous slurry. After polishing, samples were rinsed with ethanol and sonicated for 1 min in ethanol to remove any polishing residue.

### 2.2. Electron Microscopy

A field emission scanning electron microscope (SEM, FEI Teneo, Hillsboro, USA) coupled to an energy-dispersive X-ray spectrometer (EDS, 80 mm^2^ Energy+, Oxford Instruments, Abingdon, UK) was utilized to characterize the surface microstructure and corrosion morphology of all samples, as well as construct elemental composition maps of the heat-treated surfaces. SEM analyses were conducted in both secondary electron (SE) and backscattered electron (BSE) imaging modes using 10–20 keV accelerating voltages.

### 2.3. Scanning Probe Microscopy

#### 2.3.1. Ex situ Scanning Probe Microscopy (SPM)

Ex situ AFM, MFM, and SKPFM were performed under an inert argon atmosphere containing <0.1 ppm H_2_O and O_2_ using a Bruker Dimension Icon AFM housed in an MBraun glovebox (MBraun, Stratham, USA). Prior to imaging, previously polished and sonicated samples were cleaned with HPLC/spectrophotometric grade ethanol (Sigma-Aldrich, 200 proof, St. Louis, USA) using lint-free wipes (Kimtech). Following ethanol cleaning, compressed ultra-high purity nitrogen gas (Norco UHP, 99.999%) was used to dry the surface of the steel and remove any remaining surface particulates before introducing the samples into the glovebox antechamber.

Both MFM and SKPFM were performed using a dual-pass lift mode implementation in which the first pass over each scan line acquires surface topography. Upon completing the first pass, the probe then lifts off the surface to a user-defined height above the surface. This height (i.e., tip-sample separation, 100 nm in this study) remains constant throughout the second pass as the electromagnetic property of interest (i.e., Volta potential difference in the case of SKPFM or magnetic moment in the case of MFM) is measured. Surface topography was mapped using either intermittent contact (tapping) mode in the case of MFM imaging or PeakForce tapping mode (Bruker Nano, Santa Barbara, USA), which employs rapid force curve acquisition with a user-defined force setpoint (typically 2 nN here), in the case of AFM and SKPFM. In MFM, the magnetic force gradient between a magnetized Co-Cr coated AFM probe (Bruker MESP, *k* = 2.8 N/m, *f_0_* = 75 kHz, µ = 1 × 10^−13^ EMU, where 1 EMU = 1 erg G^−1^) and the surface of the material was observed during the lift mode pass. For consistency, all MFM imaging reported herein was performed with the same MESP probe, which was magnetized immediately prior to imaging with its magnetic axis perpendicular to the sample surface. In SKPFM, the Volta potential difference (VPD) between a conductive probe (Bruker PFQNE-AL, *k* = 0.8 N/m, *f_0_* = 300 kHz) and the surface was quantified by application of a DC bias to null the tip-sample electric force gradient arising from the difference in Volta potential between the probe and sample surface. VPD maps were acquired utilizing frequency modulation SKPFM [31], as described in detail elsewhere [37,38]. These VPD maps were used to predict the corrosion behavior of the samples by suggesting the cathodic and anodic sites and the relative driving force for galvanic corrosion.

SKPFM was also used to observe corrosion initiation and propagation mechanisms by carrying out intermittent imaging at well-defined intervals throughout the corrosion process. While all such imaging was carried out within the controlled environment (<0.1 ppm H_2_O and O_2_) of the argon-filled glovebox, corrosion was initiated and allowed to proceed outside the glovebox, where samples were sequentially soaked for prescribed amounts of time in a 1 M NaCl solution prepared from reagent grade NaCl (Sigma Aldrich, St. Louis, USA) and deionized (DI) water. After each time increment, samples were rinsed with DI water to remove any adhered salt, dried with UHP nitrogen, and cleaned with ultrapure ethanol using lint-free wipes. The samples were then reintroduced into the glovebox and imaged via dual-pass SKPFM. Repeated nanoscale imaging at specific recurrent locations with micron-scale positional accuracy was made possible by fiduciary marks created with a diamond tip indenter. Testing and imaging were performed ~500 µm away from the fiduciary mark to ensure results obtained were not influenced by the indent.

#### 2.3.2. In Situ SPM

To capture images of corrosion initiation and propagation in real time, in situ PeakForce tapping (topographical) AFM was also performed. In contrast to the ex situ (i.e., glovebox) SPM imaging, samples for in situ AFM imaging were mounted in a fluid cell and immersed in a 0.1 M NaCl solution under ambient atmosphere. The NaCl concentration was chosen such that it would initiate corrosion on samples at an appropriate timescale to reveal changes in topography concurrent with corrosion propagation and progression. Silicon nitride probes with a nominal tip radius of 20 nm (Bruker ScanAsyst-Fluid, *k* = 0.7 N/m, *f_0_* = 150 kHz) were used for repetitive imaging (0.5 Hz scan rate) of 10 × 10 µm^2^ areas at 512 × 512 pixel resolution, corresponding to a refresh rate of ~8.5 min to capture each image. Due to differences in time between initial immersion of each sample and the initial image capturing (driven by optimization of imaging parameters), the specific timing of subsequent images is not exact between samples. The total amount of time each sample had been exposed to the corrosive salt solution was documented at both the start and end of captured images.

#### 2.3.3. Image Processing

**Figure 1:**
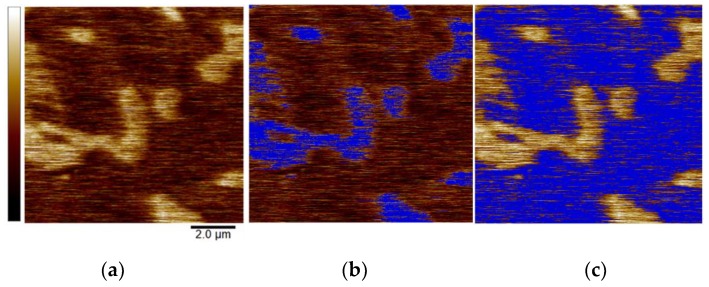
Representative 10 × 10 µm^2^ scanning Kelvin probe microscopy (SKPFM) images of P675- high-temperature temper (HTT). Dark brown corresponds to the softer matrix phase, which is lower in height following polishing than the harder, lighter brown carbides. Images show (**a**) the original Volta potential image (600 mV full-scale range) and subsequent implementation of thresholding cutoffs (blue) to calculate average Volta potential differences (VPDs) for the (**b**) matrix and (**c**) carbides.

SPM image processing and quantitative analysis were conducted using NanoScope Analysis 1.90 (Bruker). All topographical images were processed with a first order flatten filter to remove sample tip and tilt as well as any individual line-to-line offsets. The images for HTT at 116 and 135 min required a 2^nd^ order flatten procedure to account for the deposited debris. To quantify the findings from SKPFM mapping, a threshold technique was implemented (see example image in Figure 1 below) that utilized a user-determined cut-off potential based on the distribution of Volta potentials observed in the corresponding data histogram (512 bins). From the resulting thresholded data, the average Volta potential (with corresponding standard deviation) was calculated for each of the two phases present on the surface (i.e., matrix and carbides, identities confirmed through SEM/EDS characterization) [52]. Figure 1a shows a representative SKPFM Volta potential map for HTT P675. Figure 1b shows the matrix in dark brown with the carbides (data in blue) excluded, while the light brown areas visible in Figure 1c correspond to the carbides (with the matrix excluded and indicated by the dark blue areas). Using this method, an average VPD between the matrix and carbides was calculated for each SKPFM image.

### 2.4. Electrochemical Corrosion Testing

Electrochemical cyclic polarization testing was used to characterize corrosion behavior for each type of heat-treated steel. Sample preparation details can be found in a previous publication, thus the sample testing area was defined by masking off the sample such that only a circular area (diameter ~6.6 mm) test area was in contact with the electrolyte solution [7]. Testing was conducted in 0.01 M NaCl electrolyte solution with a potentiostat (SP-300, Bio-Logic, Seyssinet-Pariset, France) used to control and monitor a three-electrode system in a modified flat cell. A saturated calomel electrode (SCE) served as the reference electrode and a platinum mesh as the counter electrode. Following sample immersion, open circuit potential (OCP) was monitored for 30 min. The sample was then polarized at a scan rate of 0.5 mV/s from 100 mV below OCP to 600 mV above OCP or when pitting had stabilized, followed by a reverse scan back to OCP.

## 3. Results

### 3.1. Surface Composition

**Figure 2:**
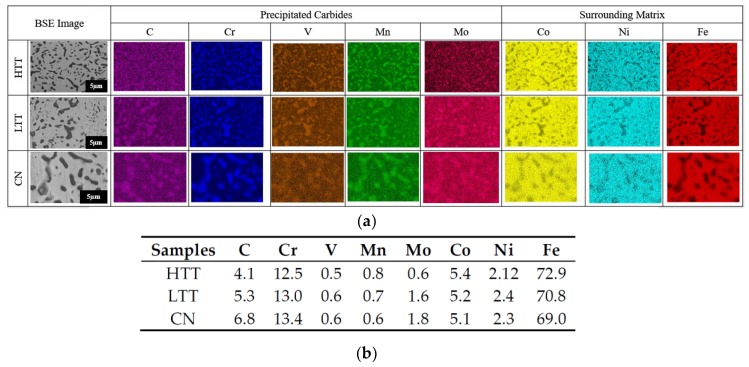
(**a**) Grayscale backscattered electron (BSE) images (left column) of the three different P675 surface-treated samples (carbides appear darker than surrounding matrix) with corresponding colored energy-dispersive X-ray spectrometer (EDS) compositional maps highlighting the principal components of the carbides (middle columns) and bulk matrix (right columns) for the HTT, low-temperature tempered (LTT), and carbo-nitrided (CN) samples (images for each row share the same micron bar). (**b**) Elemental composition in wt.% (determined via EDS) for the surface of each steel (not individual carbides).

The carburizing and carbo-nitriding heat treatment processes performed on MSS P675 resulted in the development of well distributed metal-carbon precipitates (carbides) ranging in size from approximately 10 nm to 2 µm in diameter (Figure 2a), surrounded by the martensitic matrix at the sample surface. In addition to the surface, the carbides are present diminishingly, approximately 1000 µm radially inward into each of the samples (data not shown). Sample surfaces were analyzed via EDS (Figure 2b) to resolve carbide chemistry and determine alloying elements that segregated from the matrix to form these carbides during heat treatment. Carbides resulting from all three surface treatments were found to be predominantly carbon- and chromium-rich with lesser amounts of vanadium, molybdenum and/or manganese, while the surrounding matrix showed primarily iron, cobalt, and nickel. In previous work done on P675, X-ray diffraction (XRD) and electron beam backscattered diffraction (EBSD) determined M_7_C_3_ (orthorhombic) and M_23_C_6_ (face-centered cubic) to be the primary carbides formed in P675 (M represents the metal in the carbide), with M_23_C_6_ precipitating after M_7_C_3_, and chromium being the primary metal constituent present in the carbides [27,53]. HTT contains a greater population of M_23_C_6_ carbides than LTT and CN due to its higher tempering temperature (i.e., increased kinetics). By stoichiometry, the HTT carbides contain more chromium than the carbides of the other two surface treated steels despite all having the same bulk composition before heat treating. The large amount of chromium present in the bulk (pre-heat treatment) P675 alloy (Table 1), coupled with the presence of molybdenum, should yield a magnetic MSS [54,55,56]. However, EDS analysis (Figure 2a,b), performed on the bulk surface of each steel, showed that the chromium and molybdenum primarily segregated within the carbides following heat treatment (thereby increasing the likelihood of magnetic carbides). EDS was performed on the bulk steel and not on the individual carbides due to inconsistent results obtained since large interaction volumes (by the EDS) penetrated both the carbide and surrounding matrix. In contrast, nickel, in the presence of iron and carbon acts as an austenite stabilizer and thus promotes a non-magnetic austenitic (fcc) structure [57]. MFM was therefore utilized to observe how the secondary processing performed on these steels affected the magnetic properties of the surface.

### 3.2. Scanning Probe Microscopy

#### 3.2.1. Magnetic Force Microscopy (MFM)

**Figure 3:**
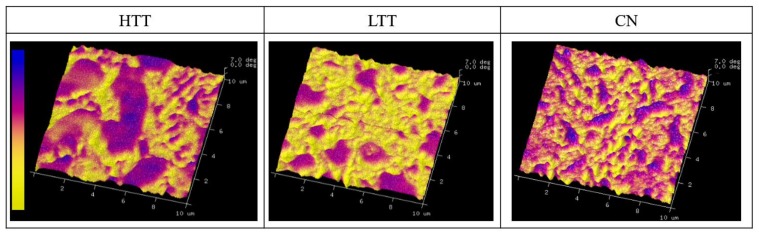
3D magnetic response maps with changes in height representative of differences in magnetism. Color scale ranges are 7 degrees (0° = yellow, +7° = blue) for magnetic response.

MFM was utilized to map variations in the magnetic moment projections (surface normal direction) on the surface of the steels (Figure 3). In Figure 3, purple regions are identified as carbides since these coincide with regions that are raised in topography and visually similar to carbides seen in SEM/EDS analysis (see Figure 2). Topographical relief of the carbides was expected due to differential polishing rates during sample prep, resulting in the harder carbides slightly protruding above the surrounding matrix. MFM results indicated that the carbides and the matrix both exhibit out-of-plane magnetic domains (i.e., positive magnetic direction, non-parallel to surface), but with varying magnitude, carbides being noticeably larger than the matrix as expected from the enhanced chromium concentration (see Figure 2). Within the matrix, nanoscale variations in magnetic domain were also evident. In HTT these were larger and more elongated those on either HTT or CN. CN had the finest distribution of different magnetic domain regions able to be resolved within the matrix.

#### 3.2.2. Inert Environment Scanning Kelvin Probe Microscopy (SKPFM)

**Figure 4:**
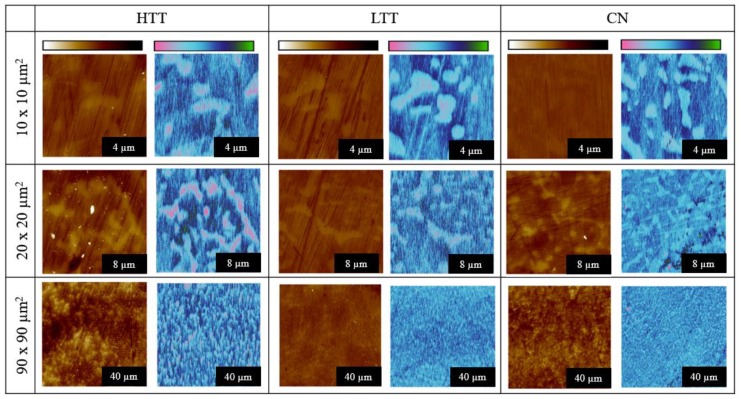
High-resolution atomic force microscopy (AFM) topography (dark brown to white color scale, 100 nm full scale) and SKPFM Volta potential (green to pink color scale, 600 mV full scale) images over different size scan areas showing the different sizes and shapes of carbides distributed throughout the three sample types.

**Figure 5:**
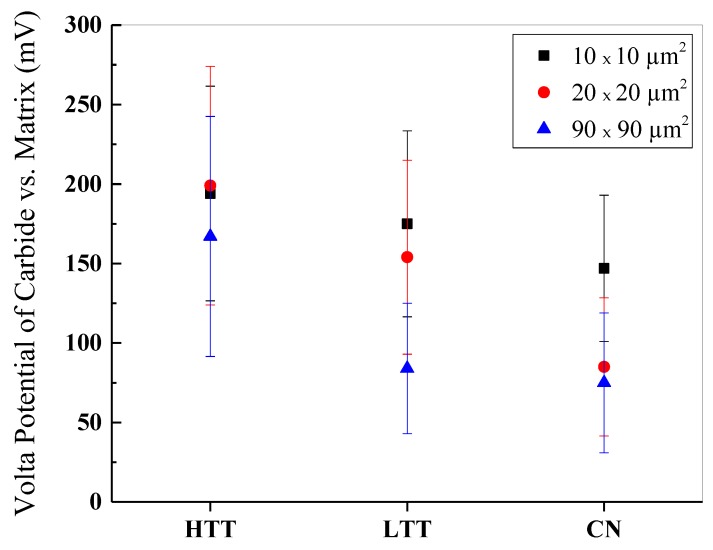
Plot of measured VPDs (with standard deviation error bars) of carbide precipitates versus the surrounding matrix for the three P675 surface-treated steels as a function of scan area.

Freshly polished, cleaned, and dried samples underwent ex situ SPM imaging in an inert atmosphere glovebox. Images were acquired using sequentially larger scan areas of 10 × 10 µm^2^, 20 × 20 µm^2^, and 90 × 90 µm^2^, with contrast between carbides and the surrounding matrix observed in both Volta potential and topography (Figure 4). Numerical VPD results were calculated per the method described earlier and compiled for comparison (see Figure 5, error bars are indicative of one standard deviation). The measured VPD of the carbides ranged from 60 to 200 mV greater than the steel matrix, depending on the scan size analyzed, with HTT possessing the highest difference and CN the lowest. The relative magnitudes of the carbide-matrix VPDs remained consistent regardless of scan size, suggesting even the smallest imaging areas chosen (10 × 10 µm^2^) were large enough to be representative of the sample while also providing the highest spatial resolution of VPD variations.

#### 3.2.3. Intermittent SKPFM

**Figure 6:**
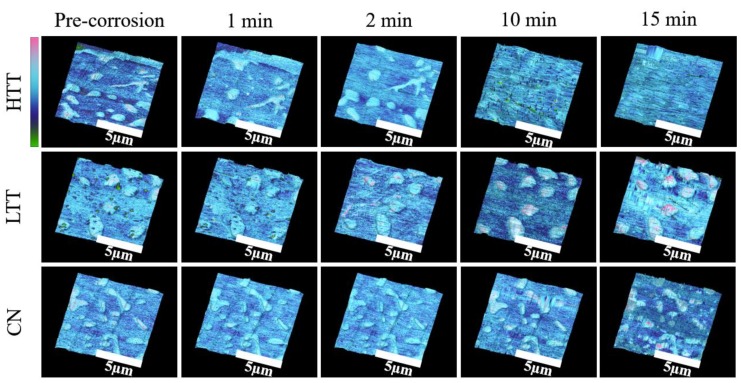
SKPFM Volta potential maps (green to pink color scale, 400 mV full scale) overlaid on the evolving 3D topography (30 nm full scale) of the three heat-treated MSSs as a function of immersion time in 1 M NaCl solution.

Intermittent ex situ SKPFM was performed to track the evolution of the surfaces resulting from sequential sustained exposure to corrosive conditions. Samples were placed in a corrosive salt solution and the VPD maps were obtained at intervals of 0, 1, 2, 10, and 15 cumulative minutes of exposure to 1 M NaCl solution (Figure 6). Qualitative differences in both appearance (surface topography and morphology) and carbide-matrix VPD over time were observed for the steels. The HTT sample showed the formation of particulates on the surface and degraded uniformly with time, leading to a progressively lower variation in surface VPD. In contrast, the CN sample showed little change in VPD or topography on the surface, indicating corrosion reaction kinetics were much slower despite the distinct VPD contrast between the carbides and matrix. LTT exhibited behavior somewhere in between the other two steels. Initially, salt deposits on the LTT surface obscured the steel topography and VPD variations. However, with increasing time LTT appeared similar to CN, as evidenced by the relatively large contrast in topography and VPD apparent by the 15 min mark (see Figure 6).

**Figure 7:**
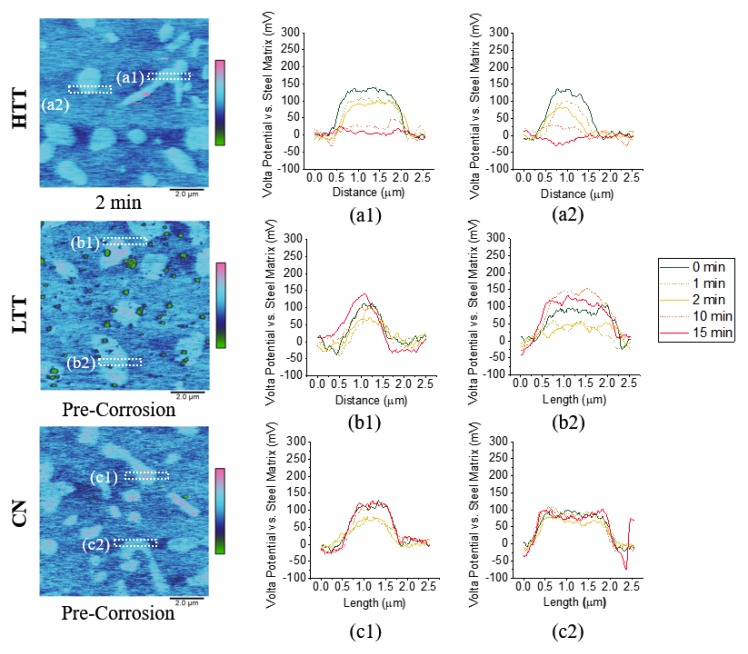
SKPFM Volta potential maps ((**a**,**b**,**c**) 600 mV full scale, exposure time given below each image) for each of the three heat-treated MSSs with time-dependent Volta potential profiles (**a1**–**c2**) across two representative carbides plotted as a function of duration of exposure to 1 M NaCl solution. The location of the carbide represented by each profile is indicated by the corresponding dotted box in the exemplary SKPFM maps at left.

Figure 7 presents VPD maps (left column) and plots of Volta potential versus location (middle and right columns) for cross sections of different carbide/matrix interfaces as a function of exposure time. As can be seen in the top row of Figure 7, the VPD between the HTT carbides and the surrounding matrix decreased with exposure time, while the VPDs of the LTT (Figure 7(b1,b2)) and CN (Figure 7(c1,c2)) carbides remained relatively constant throughout testing. For HTT, corrosion proceeded simultaneously both along grain and carbide boundaries as well as within the matrix. Corrosion products evolved and settled on both the matrix and surface carbides, where cathodic activity was supporting anodic dissolution of the matrix. With this production and deposition of corrosion products, the VPD between carbides with a native oxide and matrix decreased on the HTT surface until there was very little difference observed between the two, as seen in Figure 7(a1,a2). Conversely, the LTT and CN samples underwent typical localized corrosion (see Figure 6), wherein highly localized attack adjacent to grain boundaries/carbides was seen, as evidenced by particulates settling on or near the carbide-matrix interface. As time in solution progressed, the VPD between the carbides and steel matrix remained essentially unchanged throughout the duration of testing, with matrix attack relatively shallow. Therefore, there are notable differences in the initiation of corrosion mechanisms between different heat treated samples.

#### 3.2.4. In Situ Atomic Force Microscopy (AFM)

**Figure 8:**
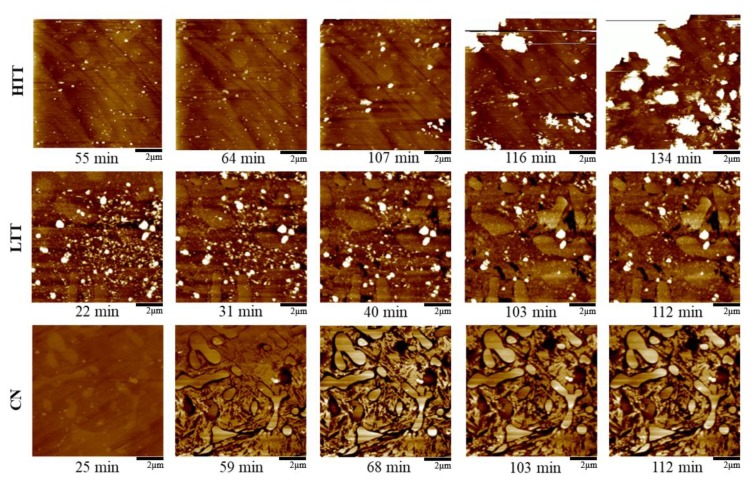
Time-lapse in situ AFM topography maps (160 nm full scale) for each of the heat-treated MSSs in 0.1 M NaCl solution, with approximate exposure time at the end of each scan indicated below the corresponding map (image time was ~8.5 min).

To observe the progression of corrosion in real time while samples were immersed in 0.1 M NaCl solution, in situ AFM was employed to monitor topographical changes over time. Figure 8 shows the results for the three heat-treated P675 steels with no applied bias voltage. (Variations in exposure time across samples are due to differences in corrosion rate and the time necessary to implement optimized imaging parameters.) For HTT, corrosion activity rapidly progressed and large surface deposits (~1–2 µm wide) appeared on the surface after ~107 min (Figure 8). EDS analysis indicated these large features to be iron-rich corrosion products with NaCl (analysis not shown). Despite the deposited particles, distinct localized corrosion was not seen on the HTT sample. As testing progressed, corrosion reactions proceeded, depositing corrosion product particulates on the surface (see Figure 8–HTT 116 & 134 min). In comparison, highly localized corrosion was evident at the carbide-matrix interfaces in both the CN and LTT samples. CN showed the greatest segregation of corrosion between matrix attack and the unaffected carbides, as indicated by near complete but shallow etching attack along carbide boundaries (Figure 8). LTT appeared to behave somewhere in the middle of these two extremes, with particle build-up similar to HTT seen initially, but eventually, these particles cleared to reveal evidence of localized corrosion propagation in the matrix adjacent to some of the carbides, similar to CN.

**Figure 9:**
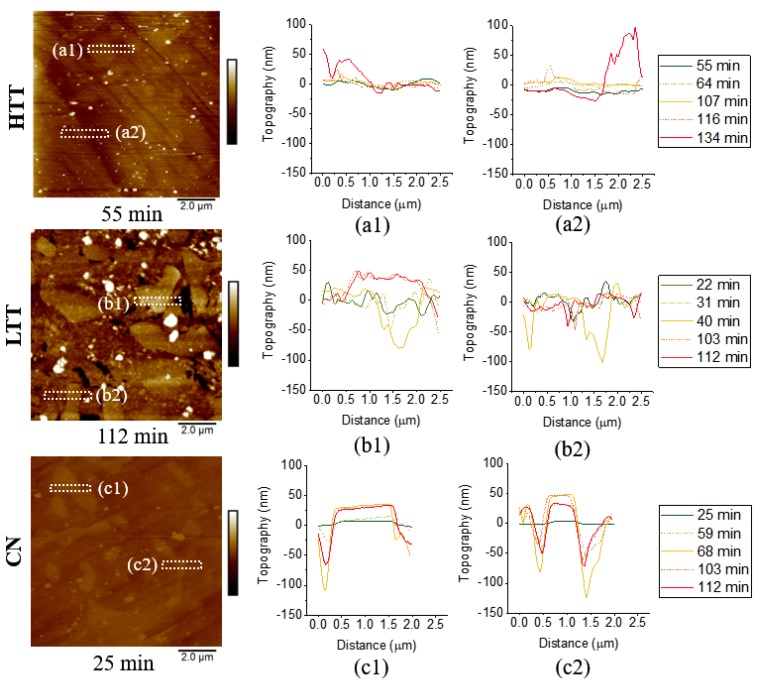
Topography maps ((**a**,**b**,**c)**, 160 nm full scale, exposure time indicated below corresponding map) for each of the three heat-treated MSSs with height profiles across selected carbide-matrix interfaces shown as a function of exposure time to 0.1M NaCl solution (**a1**–**c2**). Location of each profile is indicated by the corresponding box in the exemplary topography maps presented at left for each of the three heat-treated steels.

Time-dependent line profile analysis of selected carbide particles was conducted on each of the samples (Figure 9), confirming the qualitative observations arising from the images presented in Figure 8. HTT showed an increase in surface contrast of the carbides, up to 50 nm, with corresponding slight, uniform changes in the height of the surrounding matrix. For LTT, height changes across the carbide/matrix interface initially (44 min) showed ~100 nm deep attack immediately adjacent to the carbides (Figure 9). Then at longer times (112 min), the height of the carbides increased, accompanied by shallower apparent depth of attack in the adjacent matrix area. These changes are likely associated with the production and deposition of insoluble corrosion products. CN exhibited the sharpest contrast in topography by the end of exposure to salt solution, with the carbide surface height increasing by ~25 nm relative to the adjacent bulk matrix, with matrix attack limited to ~75 nm deep and only extending approximately 0.5 µm away from the carbide interface. The depth of attack also decreased from 103 min to 112 min, indicating slight corrosion product deposition within the highly localized area of matrix attack.

**Figure 10:**
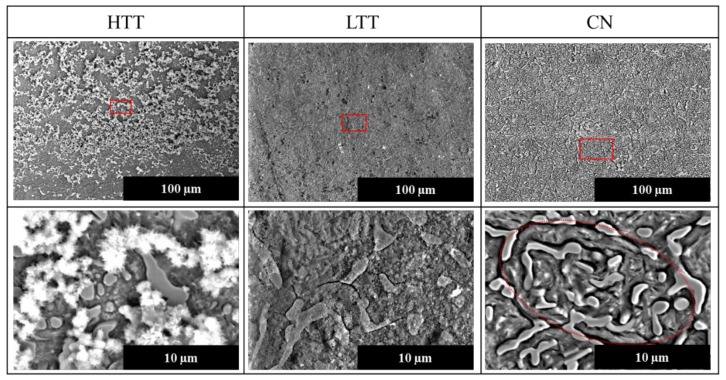
SE SEM images of the sample surfaces following in situ AFM testing. Red squares in the images in the top panels indicate areas of magnified images below. Dotted red oval area in magnified CN image indicates the “line of attack” (see discussion).

Post-testing SEM imaging was conducted on the same sample surfaces (Figure 10) to record surface morphological differences following the in situ AFM testing. HTT exhibited a distinctively different surface morphology compared to LTT and CN, characterized by the presence of large, fluffy appearing salt-laden corrosion deposits. Beneath these deposits and surrounding the carbides, the entire matrix surface area was uniformly corroded with no indication of matrix passivity. In contrast, both the LTT and CN carbide boundaries were attacked, with NaCl particles present along the grain boundaries and carbide-matrix separation and subsequent grain separation (Figure 10). LTT showed some attack along carbide boundaries as well as some generalized attack as indicated by roughening of the entire surface due to corrosion product deposition. CN displayed much more localized attack at the carbide boundaries than LTT (dotted oval in the right panel of Figure 10), and narrow “valleys” on the order of ~0.5 µm wide were observed around the CN carbides, confirming observations in Figure 8. Furthermore, unlike LTT or HTT, CN did not show evidence of adhered or deposited corrosion products. Tracing the representative “line of attack” for the CN sample in Figure 10 reveals a grain undergoing intergranular attack, indicative of microgalvanic corrosion between the noble carbides and the active matrix.

### 3.3. Electrochemical Corrosion Testing

**Figure 11:**
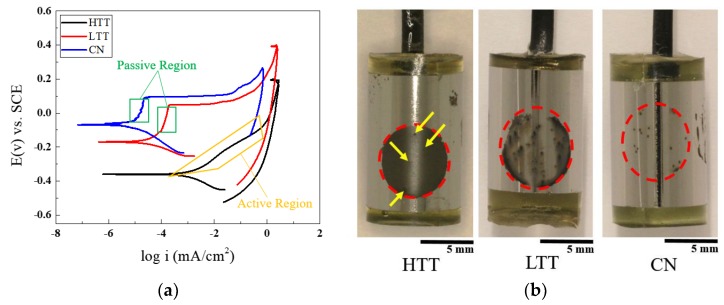
(**a**) Cyclic potentiodynamic polarization (CPP) scans (0.01 M NaCl electrolyte) for all three surface treatment samples. Passive regions for LTT and CN are indicated by green squares. (**b**) Images of the samples post-testing (after the area masking tape was removed) with dotted red circles indicating the test location on each sample surface. All samples display some isolated pitting. However, due to the difficulty in clearly seeing the pits on the HTT sample (which, in contrast to the other samples, underwent generalized corrosion attack), yellow arrows indicate the location of the pits present on the HTT sample.

To elucidate the corrosion pitting and repassivation behavior of the samples, cyclic potentiodynamic polarization (CPP) scans were conducted on each of the samples to explore the effects of the different heat treatments. Figure 11 shows the resultant polarization curves, along with macro images of the sample surfaces post-electrochemical testing. Testing indicated that HTT had the lowest OCP (−400 mV), followed by LTT (−200 mV) and CN (−80 mV), respectively. This ranking is in agreement with previous studies that ranked corrosion resistance for these same steels (i.e., corrosion rate determined via electrochemical methods) [7,9]. The LTT and CN samples exhibited a rapid change in potential over a minimal increase in current density (Figure 11a, green boxed areas), indicative of typical passive behavior. The breakdown potential of the LTT and CN samples occurred at 40 mV and 95 mV, respectively. Conversely, the HTT sample showed active corrosion behavior as demonstrated by linear growth of the current density over the potential sweep. However, pits were initially observed on the HTT surface (−200 mV), but did not grow and as the anodic overpotential continued to increase. The post-corrosion images in Figure 11b show the difference in corrosion morphology for each sample following CPP testing. For HTT, the entire test area darkened due to corrosion product formation (Figure 11b), engulfing the initially isolated areas of pitting. Arrows in Figure 11b indicate the four pits that first formed on the HTT sample before the entire test area underwent generalized corrosion. As expected from previous work [7], LTT and CN showed a distinctly different morphology of corrosion attack, with corrosion limited to only several dispersed pits on the surface of the sample. Compared to HTT, LTT showed limited regions of depassivation emanating from corrosion pits, evidenced by regions of minor surface darkening. In contrast, corrosion attack on CN displayed only highly localized, isolated pits (Figure 11b) with no visual evidence of any other associated areas of depassivation.

## 4. Discussion

### 4.1. Nanoscale Origins of Corrosion Initiation

Determining the nanoscale contributions to a material’s bulk corrosion rate is inherently difficult due to the complexity and multitude of variables that influence its behavior in a corrosive environment. Corrosion is a spontaneous process driven by thermodynamics [58,59]. In a microgalvanic couple, the difference in electrode potential of the anode and cathode regions on the surface correlates with the magnitude of negative free energy change (thermodynamic propensity) for local corrosion to occur. SKPFM Volta potential (VPD) mapping is the highest spatial resolution method available to directly measure the relative thermodynamic propensity for corrosion between nanoscale heterogeneities in a material. For the MSSs considered in this study, the relatively high Cr composition of the carbides suggests they are likely noble in comparison to the matrix based on the galvanic series [60]. Hence, a larger VPD between carbides and the matrix will lead to a greater drive (i.e., increased microgalvanic full-cell potential) for corrosion of the matrix. Among the steels studied, HTT consistently exhibited the largest VPD between the carbides and the matrix (200 mV), while LTT (150 mV) and CN (90 mV) were considerably less (Figure 5). The relative magnitudes of these VPDs can likely be attributed to carbide chemistry, as HTT carbide composition is predominantly M(Cr)_23_C_6_ compared to predominantly M(Cr)_7_C_3_ compounds for LTT and CN. An interesting finding of this study is that for each of the surface treatments considered, the bulk OCP values measured inversely corresponded with the magnitude of the VPD between the carbide and matrix phases (Figure 5). HTT had the greatest carbide/matrix VPD and least noble OCP (−400 mV), CN had the lowest VPD and most noble OCP (−80 mV), and LTT was intermediate with a carbide/matrix OCP of −200mV. This observation demonstrates how local SKPFM measurements of the relative microgalvanic couple potential contribute to the bulk OCP observed on each of the different surface-treated MSSs investigated. In addition, variations in chromium enrichment of the carbides subsequently influenced both the VPD and degree of passivity of the surrounding chromium-depleted matrix. The steepest VPD gradients measured were across the carbide/matrix interface (Figure 7), and so SKPFM measurements also provided a technique to predict and locate expected points of microgalvanic corrosion initiation on the surface.

### 4.2. Corrosion Propagation

SKPFM measures VPDs on the surface, which are influenced by the presence of oxide layers. With MSSs, passivating chromium oxide layers are readily formed and act as a kinetic barrier to corrosion, which complicates any correlation of thermodynamic propensity derived from SKPFM measurements. However, for the steels considered herein, since the bulk composition is the same, data obtained from SKPFM also provided information on the spatial variations in surface properties that influence corrosion propagation. Intermittent SKPFM testing was conducted to monitor shifts in microgalvanic couples’ VPD over time due to corrosion activity. For HTT, the VPD between the carbides and the matrix decreased with time (Figure 7). As a result, as the duration of corrosion propagation increased, the VPD between carbides and the matrix approached 0 mV for HTT, resulting in a more thermodynamically homogenous surface. In contrast, for LTT and CN, the initial VPD between the carbides and matrix phase was smaller, but remained nearly constant throughout testing, with only minor evidence of the corrosion activity apparent on the surface (Figure 6 and Figure 7). This behavioral difference can be attributed to differences in the passive oxide layer performance, and is also reflected in the VPD measurements, which are highly influenced by the presence of surface oxides. Previous work by Schmutz and Frankel showed similar behavior on aluminum alloys and indicates that the shift in VPD observed on HTT following active corrosion was caused by oxide growth at cathodic sites and the generation and deposition of corrosion products at active sites creating a more homogenous surface [51]. For carburized MSSs, the magnitude of VPD surface variation measured by SKPFM pre-corrosion provided an indication of the how the VPD evolved as a result of exposure to corrosion conditions: smaller initial VPD between the carbides and matrix phase indicated more robust passivity during corrosion, as seen in CN and LTT steels. For HTT, the higher initial VPD between the carbides and matrix indicated a greater susceptibility to depassivation and more uniform corrosion activity during propagation. These findings were validated with bulk electrochemical testing (Figure 11), where CPP testing showed that LTT and CN had a more protective oxide layer as indicated by the presence of a passive region in the CPP scan. Moreover, during intermittent SKPFM testing the VPD on HTT evolved rapidly and HTT exhibited active corrosion behavior throughout CPP testing.

While the bulk amount of chromium present at the surface is the same for all steels considered, the spatial distribution is different among the three surface treatments, leading to distinctly different corrosion properties and behavior. Relative to LTT and CN, HTT tended to corrode more uniformly and had a higher VPD between carbides and matrix. HTT was more prone to depassivation compared to LTT despite both having identical bulk chemical composition and same carburization cycle (carburized in single furnace load). The different carbide-matrix VPDs among the samples influences or indicates how local solution chemistry likely evolves during active corrosion on MSSs. This suggests that for HTT, as pitting progressed, the local solution chemistry, most likely due to higher sensitization during tempering cycle, was sufficiently aggressive to cause widespread depassivation. Conversely, with LTT and CN samples, the VPD between carbides was smaller and pitting was unable to transition to more widespread corrosion, suggesting local solution chemistry evolution did not support auto-catalytic depassivation as corrosion propagated. Here the lower VPD observed for LTT and CN indicated the matrix phases exhibited more robust passivity than the matrix of HTT. The in situ SKPFM VPD measurements correlate with the observed corrosion morphology of the steels. That is, the measured carbide-matrix VPD for each steel is inversely proportional to the extent of general (uniform) corrosion resistance of the steel. The efforts in this paper show that SKPFM is able to effectively predict bulk corrosion behavior of different surface treatments by observing and measuring nanoscale surface VPD differences between carbides and the underlying matrix.

### 4.3. SPM Characterization and Implications on Wear

MFM provides a method to characterize local variations in magnetic properties that contribute to the bulk magnetic properties. For all steels studied, the carbides showed variable shades of purple/blue in the MFM maps (~1–3° phase shift), indicating slightly different magnetic properties within the individual phases (Figure 3), likely due to different carbide compositions in terms of the relative amounts of chromium and molybdenum, which influence the magnetic properties of phases [61,62,63,64]. Sample CN had a much less homogenous matrix that showed considerable variation in magnetic properties and is likely an effect of the relatively high surface retained austenite (18–22%) found within the matrix phases compared to LTT (10–13%) and HTT (1–2%) [9,30]. The bulk magnetism of the steels will change with tempering temperature and heat treatment process, following changes to the microstructural phases formed [9,30]. Further work is currently underway to investigate the implications of local magnetism and magnetic domains on resulting wear and corrosion mechanisms.

**Figure 12:**
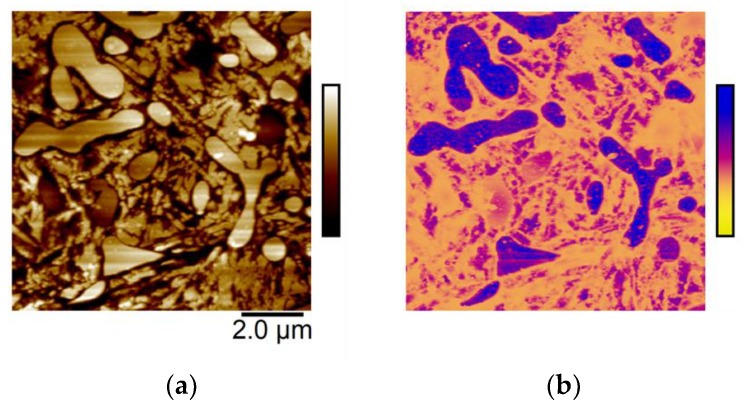
(**a**) CN topography (160 nm full scale), and (**b**) DMT Modulus (1.5 GPa full scale). Images are representative of 103–112 min submersion in 0.1M NaCl solution.

Similarly, the ability to resolve nanoscale variations in the resistance to deformation (elastic modulus) on a material’s surface could help improve prediction of the wear behavior. The PeakForce tapping mode employed here measured differences in the elastic modulus distribution, as determined via the Derjaguin-Muller-Toporov (DMT) model [65], for the carbide and the matrices of the steels simultaneously with topography (see exemplary Figure 12). As seen in the CN image presented in Figure 12, carbides had a higher relative modulus than the matrix, suggesting potential sites for development of micro-cracking and fracture would likely lie at the interface between carbides and matrix where local modulus variation was greatest. Further work is underway to determine how these local differences in recorded modulus correlate to a material’s ability to handle loads/stress in bearing applications.

In service, the uniform degradation seen on HTT could be effectively monitored conventionally via visual inspection, detection of wear debris, or thickness monitors installed on bearing raceways. For CN and LTT, current methods of monitoring engine health are less effective since significantly lower amounts of reaction products are generated from highly localized corrosion. Localized corrosion may not be detected until it has led to significant wear damage. Bearing steel developers should, therefore, be cautious with heat treatments that yield a surface similar to CN which, although highly corrosion resistant, the passive surface will inevitably be compromised in wear applications. Small areas of highly localized corrosion pits lead to surface crater development which can potentially lead to highly undesirable and unpredictable failure via spalling. LTT behavior was intermediate between the two other surface treatments, with some localized attack on grain/carbide boundaries as well as some evidence of wider depassivation. In corrosive environments, the overall wear lifetime may be controlled by resistance to corrosion initiation, in which case LTT and CN could provide greater benefit than HTT. Previously conducted wear studies are in agreement with the recommendations given, and the results of this study provide nanoscale insight to help understand why HTT outperformed both CN and LTT during rolling contact fatigue testing even though it had significantly lower corrosion resistance [29,30]. Based on this work, P675 HTT would be recommended over the other two tempering procedures for use in aerospace bearings where corrosion is not a primary concern. However, when the bearing assembly is prone to corrosion attack, HTT is not recommended due to its overall low corrosion resistance [7] which would lead to premature failure via degradation of the material. In this case, CN is recommended for bearing use due to its high resistance to both corrosion onset and propagation [7].

## 5. Conclusions

P675 carburizable martensitic stainless steel (UNS S42670) samples were processed using two different heat treatment methods (carburizing and carbo-nitriding (CN)) and two tempering temperatures (HTT and LTT). Following, the research conducted in this paper highlights the viability of SKPFM to effectively predict bulk corrosion behavior by measuring nanoscale surface differences in VPDs between carbides and the surrounding matrix, thereby providing insight into bulk observations by using information obtained at the nanoscale. More generally, SPM can be used to evaluate the potential efficacy of different steels and/or surface treatments for use in corrosive environments.

- MFM imaging distinguished local differences in magnetic properties where precipitated carbides exhibited a larger magnetic moment than the matrix, likely due to the presence of chromium relative to the chromium-depleted matrix.
- SKPFM VPD measurements in an inert environment showed HTT as the thermodynamically most favorable to experience microgalvanic corrosion between the chromium-rich precipitated carbides and the surrounding martensitic matrix, with a measured carbide-matrix VPD of 200 mV, while LTT (150 mV) and CN (90 mV) were less.
- Intermittent SKPFM showed the HTT sample behaved differently during corrosion than the LTT and CN samples; by the end of the testing period, there was minimal VPD between the HTT carbides and the surrounding matrix, whereas the carbides present in the LTT and CN samples retained their relative nobility throughout testing.
- Corrosion propagation was also monitored in real time via in situ AFM and revealed that HTT underwent the most rapid spread of corrosion attack across the sample, while LTT and CN were less affected and showed much more localized, intergranular attack and adjacent to carbides.
- Bulk electrochemical testing results agreed with in situ AFM results, with LTT and CN showing distinct passive regions as compared to HTT, confirming the nanoscale differences in corrosion behavior observed between the steel heat treatments investigated.

## Figures and Tables

**Table 1:** Nominal composition (wt.%) of P675 alloy (remainder is Fe). Adapted from Trivedi, et al. [29].

Steel	C	Mn	Cr	Mo	Si	Ni	S	V	Co
P675 (AMS 5930B)	0.07	0.75	13	2	0.4	2.5	0.010	0.6	6.5
